# Multi-Section Magnetic Soft Robot with Multirobot Navigation System for Vasculature Intervention

**DOI:** 10.34133/cbsystems.0188

**Published:** 2024-11-28

**Authors:** Zhengyang Li, Qingsong Xu

**Affiliations:** Department of Electromechanical Engineering, Faculty of Science and Technology, University of Macau, Macau, China.

## Abstract

Magnetic soft robots have recently become a promising technology that has been applied to minimally invasive cardiovascular surgery. This paper presents the analytical modeling of a novel multi-section magnetic soft robot (MS-MSR) with multi-curvature bending, which is maneuvered by an associated collaborative multirobot navigation system (CMNS) with magnetic actuation and ultrasound guidance targeted for intravascular intervention. The kinematic and dynamic analysis of the MS-MSR’s telescopic motion is performed using the optimized Cosserat rod model by considering the effect of an external heterogeneous magnetic field, which is generated by a mobile magnetic actuation manipulator to adapt to complex steering scenarios. Meanwhile, an extracorporeal mobile ultrasound navigation manipulator is exploited to track the magnetic soft robot’s distal tip motion to realize a closed-loop control. We also conduct a quadratic programming-based optimization scheme to synchronize the multi-objective task-space motion of CMNS with null-space projection. It allows the formulation of a comprehensive controller with motion priority for multirobot collaboration. Experimental results demonstrate that the proposed magnetic soft robot can be successfully navigated within the multi-bifurcation intravascular environment with a shape modeling error $3.62\pm 1.28^{\circ }$ and a tip error of 1.08±0.45mm under the actuation of a CMNS through in vitro ultrasound-guided vasculature interventional tests.

## Introduction

In recent decades, soft continuum robots (SCRs) have been extensively developed due to their flexibility and rapid response, promoting their wide applications in minimally invasive surgery [1]. Especially, in cardiovascular interventional surgery, the conventional method is to utilize a manually steered cable-driven catheter to deliver the drugs/stent to the lesion region of interest (ROI) (such as thrombus and aneurysm) under the guidance of x-ray fluoroscopy imaging. This method requires surgeons to have high manipulation skills, and both surgeons and patients will suffer radiation damage [2]. To address the aforementioned drawbacks of manually steered methods, autonomous soft robot-assisted intravascular steering methods have been proposed in the literature [3]. However, cable-driven SCRs still exhibit several limitations, e.g., hysteresis and cumbersome structures, that prohibit them from more challenging scenarios in cardiovascular treatment [4].

Magnetic soft robots (MSRs) [5–7] can be advantageous over cable-driven SCRs due to their miniaturization and noncontacting teleoperated magnetic control [8,9]. To take advantage of the characteristics of MSRs, researchers attempted to apply MSRs to intraluminal and endovascular surgical procedures, e.g., cardiac ablation [10], embolization therapy [11], bioprinting [12], colonoscopy [13], and drug delivery [14]. However, conventional MSRs suffer from the drawbacks of single curvature and the incompetence of challenging multi-bifurcation steering. To address these issues, a more versatile and composite design of the MSRs is necessary to adapt to different surgical requirements [15].

Novel conceptional design of MSRs has recently emerged to facilitate new requirements for minimally invasive surgery. These new MSRs attempt to modify the structure of the single-tube shape configuration of the MSR to enhance the clinically relevant functionality, e.g., multi-section [16], concentric tube [17], variable stiffness [18], and helical shape [9]. The multi-section MSRs have the capability to deform as multi-curvature shapes to traverse through the human body lumen [19], which shows high potential in adapting to anatomical variabilities. However, the state-of-the-art multi-section MSRs lack the telescopic motion to steer at different tube configurations. Inspired by concentric tube robots (CTRs), this work aims to design a novel multi-section MSR (MS-MSR) with telescopic motion to form various shape configurations. The elastic instability indicates that conventional CTR only suits short-term transmission surgical tasks. For intravascular intervention [14], the navigation procedure is relatively long (e.g., from femoral entry to the heart). Targeted for intravascular steering with multi-bifurcation scenarios, it is desirable to take advantage of the structure of CTRs to develop a novel MSR with softer material and telescopic concentric tube structure to fit the intravascular environment while eliminating the limitations of the conventional ones. On the other hand, in clinical-related applications, it is crucial to predict the shape of SCRs within the human body, which correlates with the kinematic and dynamic responses of MSRs. The inverse kinematics (IK) modeling can be expressed using the theory of piece-wise constant curvature (PCC) [20], Cosserat rod theory [21], and Euler–Bernoulli theory (E-B) [22], etc. When external loads are considered, e.g., magnetic wrench, PCC and E-B are inaccurate in ascribing to discretized and dynamic intravascular applications. Considering the multi-section characteristic, the composite curvature and workspace of the desired MSR should be addressed in consideration of optimization [23].

Magnetic navigation systems (MNSs) [14,24] are important magnetic field generation sources for the MSRs’ actuation. They are generally classified into 2 categories, i.e., stationary MNS (sMNS) [25] and mobile MNS (mMNS) [24]. sMNS utilizes multiple fixed electromagnetic coil/permanent magnets to actuate magnetic robots, especially micro-robots [26,27]. However, such an actuation system suffers the drawbacks of limited workspace and relatively large scale when applied in the surgical field. mMNS has a more promising application due to its long-range motion, high dexterity, and movability [28,29]. However, the existing mMNS are common with a single-armed configuration that lacks cooperation and synchronization with external tracking devices [30]. Meanwhile, a multirobot configuration of surgical robots has proved successful in many surgical applications [31,32]. These surgical robotic systems integrate distinct surgical tools and medical imaging devices to the end-effector (EE) of multiple manipulators. Nevertheless, the control of a collaborative multirobot navigation system (CMNS) for endovascular catheterization remains challenging due to the redundancy of multiple degree of freedom (DOF) and a fusion of multiple actuation units, including the magnetic wrench deflection, the MSR’s insertion motion, and real-time instrument tracking. The kinematics and dynamics of the multi-objective collaborative systems require optimization to improve the planning and control accuracy in the task space [33].

The instrument tracking to localize the motion of the MSR within the patient’s body is another crucial topic in implementing remotely autonomous surgical solutions. In the current intravascular interventional surgery, x-ray [34], electromagnetic tracking (EMT) [35], and ultrasound (US) [36] are the most reliable and effective instrument tracking methods. Among these, the US is the most promising technology that could realize an autonomous robotic solution and provide both instrument and anatomical information to the surgeon. Autonomous robotic US systems for vasculature detection have been proposed in the literature [37], and they are utilized to detect the ROI within the US imaging frame. Also, the impedance modeling of the mobile US probe and the contacting tissue should be modeled to reach stable US imaging sequences [38].

This paper proposes comprehensive analytical modeling of a multi-section structured MSR with a CMNS. For the first time, such MSR and CMNS are modeled and validated for intravascular intervention. Overall, this work’s main contributions are presented as follows.

1. A novel MS-MSR navigated by a CMNS for intravascular steering with US guidance.
2. An optimized Cosserat rod theory-based kinematic and dynamic modeling of MS-MSR is constructed with the optimal planning of an external heterogeneous magnetic field.
3. A quadratic programming (QP)-based optimization scheme to synchronize the hierarchical motion of CMNS.

To address the aforementioned challenges, we first describe the design and prototyping of MS-MSR and the collaborative multirobot surgical system. Additionally, the kinematics and magnetic modeling of MS-MSR are carried out to facilitate the curvature computation during the steering. Then, we present the analytical modeling and QP-based method of CMNS. The experimental studies were performed to validate the effectiveness of MS-MSR, including in vitro experiments on a vasculature phantom.

## Materials and Methods

### Design and prototyping

**Fig. 1.**
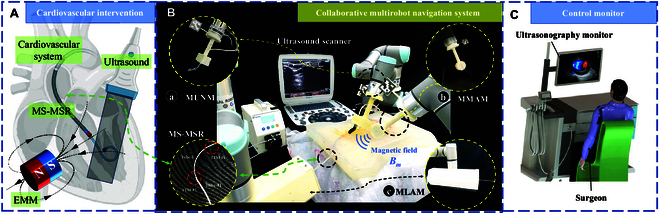
(A) Schematic illustration of MS-MSR in US-guided cardiovascular intervention, which is constituted of 2 cannulas with integrated tip magnets. (B) Design details of CMNS. MS-MSR is steered into the patient’s body through the main artery of the thigh by the corresponding actuation and tracking system. (a) The mobile US navigation manipulator (MUNM) with US probe contacts the body of the patient to enable real-time tracking of the MS-MSR’s tip. (b) The mobile magnetic actuation manipulator (MMAM) utilizes a UR5e robotic manipulator to mount an external mobile magnet (EMM). (c) The mobile linear advancement manipulator (MLAM) is advancing MS-MSR inside the artery. (C) The surgeon could remotely control the entire surgical system through the US monitor.

The primary targeted application scenarios of MS-MSR and CMNS are catheter-assisted therapy for cardiovascular diseases, e.g., stenosis and cardiac arrhythmias, as shown in Fig. 1A. The major challenge of cardiovascular intervention is the anatomical variability with multiple bifurcations connecting the heart and aorta artery. The proposed MS-MSR could overcome this difficulty by telescoping the suitable tube considering the inner diameter (ID) and curvature of the targeted blood vessel. Unlike conventional x-ray-guided intravascular intervention, we exploit ultrasonography to track MS-MSR and the vicinity to reduce radiation harm. CMNS has 3 main components (see Fig. 1B), namely, (a) MUNM: the mobile US navigation manipulator integrates a US probe and a force/torque sensor to the distal end of a UR3e serial manipulator (Universal Robots, Odense, Denmark) to realize the MS-MSR tracking and constant-force interaction model; (b) MMAM: the mobile magnetic actuation manipulator integrates a cylindrical axially magnetized permanent magnet on the EE of a UR5e serial manipulator to provide a rotating external magnetic field **B**_m_; (c) MLAM: the mobile linear advancement manipulator is an advancement module embedded with linear actuation units to realize the telescopic motion of MS-MSR using 2 separate lead screw drivers, which is installed on the EE of a 6-DOF UR3e serial manipulator. CMNS is deployed within the operating room, and the surgeons can remotely inspect the intraoperative US images and remotely control CMNS to function correctly while tracking the position of MS-MSR (see Fig. 1C).

**Fig. 2.**
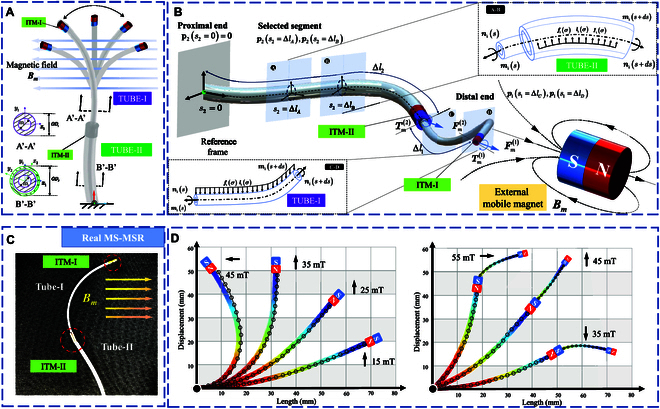
Modeling of the kinematics and dynamics of MS-MSR under different telescoping states based on the Cosserat rod theory. (A) Design details (including cross-section) of the deformation of 2 PDMS tubes under the external magnetic field B_m_. (B) Deflection kinematics of MS-MSR w.r.t. the arc length *s* considering the external loads and boundary conditions. The forces and moments acting on an infinitesimal segment of MS-MSR: 2 tubes (right top) and single tube (left bottom). (C) Actual photo of MS-MSR under the effect of the external magnetic field. (D) Comparisons of FEM results and numerical computation results of MS-MSR under different magnetic field strengths in 2 states: single tube (left) and 2 tubes (right).

MS-MSR is designed to facilitate the complex endovascular steering environment under the actuation of an external magnetic field $B_{m}$, as shown in Fig. 2A. MS-MSR is composed of *N* segments (*N* = 2), i.e., Tube-I and Tube-II. The cannula tubes are made of polydimethylsiloxane (PDMS) material, which has a soft interaction with the vascular wall. Besides, the outer diameter (OD) of Tube-I and Tube-II are 1.8 and 3.5 mm, respectively. The specifications of the tubes are listed in Table 1. Considering the material density of PDMS as $\rho \in {\mathbb{R}}^{+}$, the second moment of area tensor $J_{i}$ is defined as the *i*th tube cross-section expressed in the body frame:

$$J_{i}=\left[\begin{array}{ccc} I_{\mathrm{xx},i} & 0 & 0\\ 0 & I_{\mathrm{yy},i} & 0\\ 0 & 0 & I_{\mathrm{zz},i} \end{array}\right]$$ (1)

**Table 1.** Specifications of the multi-section magnetic soft robot

Specifications	Value		Unit
	Tube-I	Tube-II	
Outer diameter $\left({\mathrm{OD}}_{i}\right)$	1.8	3.5	[mm]
Inner diameter $\left({\mathrm{ID}}_{i}\right)$	1.2	2.5	[mm]
Tube length $\left(\Delta l_{i}\right)$	300	250	[mm]
Density $\left(\rho_{i}\right)$	0.965	0.965	[g/ml]
Young’s modulus $\left(E_{i}\right)$	2.4	2.4	[MPa]
Momentum of inertia $\left({\mathrm{EI}}_{i}\right)$	0.41	5.45	[mm^4^]

where $I_{\mathrm{xx},i},I_{\mathrm{yy},i},I_{\mathrm{zz},i}$ are the second moment of inertia of the *i*th tube. As shown in Fig. 2A, the cross-sectional configuration of 2 different scenarios of the tube combinations is presented as *A*’-*A*’, and *B*’-*B*’, respectively. Then, the term $I_{\mathrm{xx},i},I_{\mathrm{yy},i},I_{\mathrm{zz},i}$ of the *i*th tube can be calculated as a hollow circular rod as:

$$I_{i}=I_{\mathrm{xx},i}=I_{\mathrm{yy},i}=\frac{1}{2}I_{\mathrm{zz},i}=\frac{\pi }{64}\left({\mathrm{OD}}_{i}^{4}-{\mathrm{ID}}_{i}^{4}\right)$$ (2)

where ${\mathrm{OD}}_{i}$ and ${\mathrm{ID}}_{i}$ are the outer diameter and inner diameter of the *i*th tube. There is an axially magnetized internal tip magnet (ITM) integrated into each tube’s distal end to deflect the corresponding section under the actuation of an external magnetic field $B_{m}$ as shown in Fig. 2C.

### Analytical modeling of MS-MSR

This section derives the kinematic and dynamic models of MS-MSR while considering the interaction of the heterogeneous magnetic field generated by the external mobile magnet (EMM). As MS-MSR is targeted for intraoperative application, it requires an accurate, fast, and numerically stable method capable of solving large deflections, contacts, and friction. Considering the computational efficiency while performing intraoperative modeling, we adopt the Cosserat rod theory to model the shape characteristic of the proposed MSR. In terms of optimized control of a slender rod or beam as MS-MSR, the potential energy is minimized to find the optimal solution [39], which would be achieved by combining the Cosserat rod model.

In the Cosserat rod theory, the proposed MSR is considered as a set of infinitesimal rigid cross-sections along its centerline of the arc length s, as shown in Fig. 2B. For assumption, the kinematics of MS-MSR is defined by fixing the proximal end $p_{i}\left(s_{i}\right)=0,s_{i}=0$. We define the distance from the ith tube’s distal tip to the proximal end as $\Delta l_{i}\left(i=1,\;2\right)$. As shown in Fig. 2B, MS-MSR can be simplified as a multi-section Cantilever beam, as the proximal end of the soft robot is fixed at the reference frame and remains a distal free-end motion at the ith tube’s tip. According to the Cosserat rod model equation for the soft robot beams, we define the curvature $u_{i}$ as a function of orientation (in the task space) at an arc length $s_{i}$ where $s_{i}\in \left[0,\;\Delta l_{i}\right]$ and the rate of change of position of the ith tube is expressed as $u_{i}={\left(R_{i}{\left(s\right)}^{T}R_{i}\left(s\right)\right)}^{\vee }$, in which $R_{i}\left(s\right)\in \mathrm{SO}\left(3\right)$ is the rotation matrix of the ith tube. We define the 4×4 homogeneous matric of the body frame and the ith tube of MS-MSR as follows:

$$g_{i}\left(s\right)=\left[\;\begin{array}{cc} R_{i}\left(s\right) & p_{i}\left(s\right)\\ 0^{T} & 1 \end{array}\right].$$ (3)

Here, we define $u_{i}\left(s\right)$ and $v_{i}\left(s\right)$ as the translational and rotation local changes of an infinitely small section of MS-MSR (see Fig. 2B). Then, the arc derivatives of state variables of MS-MSR are expressed as the ordinary differential equations that rely on externally distributed forces and moments with respect to (w.r.t.) the arc length s. They are expressed as follows:

$${\overset{\cdot }{p}}_{i}\left(s\right)=R_{i}\left(s\right)v_{i}\left(s\right)$$ (4)

$${\overset{\cdot }{R}}_{i}\left(s\right)=R_{i}\left(s\right){\hat{u}}_{i}\left(s\right)$$ (5)

To obtain an expression for the distributed loading, we consider the force/moment balance for a selected segment of the soft robot, as shown in Fig. 2B. The total internal forces $n\left(s\right)=\sum_{i=1}^{N}n_{i}\left(s\right)$ and moments $m\left(s\right)=\sum_{i=1}^{N}m_{i}\left(s\right)$ act in the opposite direction on both cutting surfaces of the rod w.r.t. $s_{i}$, where $n_{i}\left(s\right)$ and $m_{i}\left(s\right)$ are internal force vectors of the ith tube. Then, the equilibrium of forces and moments in the MS-MSR’s base frame can be expressed as:

$$n_{i}\left(s_{i}=\Delta l_{B}\right)-n_{i}\left(s_{i}=\Delta l_{A}\right)+\int_{\Delta l_{A}}^{\Delta l_{B}}\left[f_{i}\left(\sigma \right)+I_{i}\left(\sigma \right)\right]\mathrm{d\sigma}=0$$ (6)

$$\begin{array}{c} \begin{array}{c} \begin{array}{c} \begin{array}{c} \begin{array}{l} m_{i}\left(s_{i}=\Delta l_{B}\right)-m_{i}\left(s_{i}=\Delta l_{A}\right)+p_{i}\left(s_{i}=\Delta l_{B}\right)\times n_{i}\left(s_{i}=\Delta l_{B}\right)-p_{i}\left(s_{i}=\Delta l_{A}\right)\\ \times n_{i}\left(s_{i}=\Delta l_{A}\right)+\int_{\Delta l_{A}}^{\Delta l_{B}}\left(p_{i}\left(\sigma \right)\times \left(f_{i}\left(\sigma \right)+I_{i}\left(\sigma \right)\right)+l_{i}\left(\sigma \right)\right)\mathrm{d\sigma}=0 \end{array} \end{array} \end{array} \end{array} \end{array}$$ (7)

where the distributed forces and torques that act along the ith tube are denoted by $f_{i}\left(\sigma \right)$ and $l_{i}\left(\sigma \right)$, where σ is a dummy variable for integration. $I_{i}\left(\sigma \right)$ denotes the interaction forces between adjacent tubes, as shown in Fig. 2B. As for segment A-B (top right of Fig. 2B), the interaction forces exist and increase alongside the arc length $s_{i}$. Concerning segment C-D (bottom left of Fig. 2B), i.e., Tube-I, the interaction force is zero ($I_{i}\left(\sigma \right)=0$). By taking the partial derivative of Eqs. 6 and 7 w.r.t. the arc length s, we can obtain the equilibrium differential equations of MS-MSR below.

$$\left\{\begin{array}{c} {\overset{\cdot }{n}}_{i}\left(s\right)=-f_{i}\left(s\right)-I_{i}\left(s\right)\\ {\overset{\cdot }{m}}_{i}\left(s\right)=-{\overset{\cdot }{p}}_{i}\left(s\right)\times n_{i}\left(s\right)-l_{i}\left(s\right) \end{array}\right.$$ (8)

Considering the state variables of MS-MSR as a slender rod, we define the state variables of the *i*th tube as $\xi_{i}=\left\{n_{i}\left(s\right),\;m_{i}\left(s\right),\;q_{i}\left(s\right),\;\omega_{i}\left(s\right)\right\}$, where $q_{i}\left(s\right)≔{R_{i}}^{T}p_{i,t}\left(s\right)$ and $\omega_{i}\left(s\right)≔{\left(R_{i}^{T}R_{i,t}\right)}^{\vee }$ are the velocity and linear velocity in the local frame of the ith tube. In the linear elastic scenario, the deflection of the MSR is governed by the external force loading and corresponding material characteristics. By utilizing Eqs. 4 and 5 combining the forces and moments, we can derive the following expressions:

$$v_{i}=K_{\mathrm{se}}^{-1}R^{T}n_{i}+{\hat{v}}_{i}\left(s\right)$$ (9)

$$u_{i}=K_{\mathrm{bt}}^{-1}R^{T}m_{i}+{\hat{u}}_{i}\left(s\right)$$ (10)

where ${\hat{v}}_{i}\left(s\right)$ and ${\hat{u}}_{i}\left(s\right)$ denote the initial configuration of the *i*th tube of MS-MSR. Matrices $K_{\mathrm{se}}=\mathrm{dig}\left({\mathrm{GA}}_{i},\;{\mathrm{GA}}_{i},\;{\mathrm{EA}}_{i}\right)$ (shear and extension) and $K_{\mathrm{bt}}=\mathrm{dig}\left({\mathrm{EI}}_{x,i},\;{\mathrm{EI}}_{y,i},\;{\mathrm{EI}}_{z,i}\right)$ (bending and twist) denote the stiffness terms for MS-MSR with the shear modulus G, Young’s modulus E, and cross-sectional area $A_{i}=\left\{A_{1},\;A_{2}\right\}$ of the *i*th tube and second moment of area $I_{i}$ of MS-MSR as expressed by Eq. 2, respectively. Different from cable-driven SRs, the dynamic responses of an MSR are much faster. As for the distal tip of MS-MSR, whose motion is constrained by translation length and bending angle, the partial differential equations (PDEs) of a dynamic Cosserat rod w.r.t. the state variables $\xi_{i}$ are summarized as follows:

$$n_{i}\left(s\right)=\rho AR_{i}\left(s\right)\left({\overset{⌢}{\omega }}_{i}q_{i}+q_{i}\left(t\right)\right)-f_{i}$$ (11)

$$m_{i}\left(s\right)=\partial_{t}\left(R_{i}\rho J_{i}\omega_{i}\right)-p_{i}\left(s\right)n_{i}\left(s\right)-l_{i}\left(s\right)$$ (12)

$$q_{i}\left(s\right)=v_{i}(t)-{\hat{u}}_{i}q_{i}+\hat{\omega }v_{i}\left(s\right)$$ (13)

$$\omega_{i}\left(s\right)=u_{i}\left(t\right)-{\hat{u}}_{i}\omega_{i}$$ (14)

where $\hat{\cdot }$ denotes the mapping from ${\mathbb{R}}^{3}$ to $\mathrm{SO}\left(3\right)$. The state variables are the functions of time interval t and reference arc length s. The shape and tip estimation of MS-MSR (exerted by magnetic forces, torques, and gravity) is obtained by the statics and dynamics, which can be solved as a boundary value problem (BVP). Here, we set BVP using $p_{i}\left(t,\;0\right)=0$ and $q_{i}\left(t,\;0\right)=\omega_{i}\left(t,\;0\right)=0$.

While the aforementioned approach is more effective in finding solutions that predict a desired shape of MS-MSR under external loads, i.e., the magnetic wrench $W_{m}$, it also exhibits the disadvantage of generating multiple solutions. The principle of minimum potential energy shows that the system will be in stable equilibrium to find an optimal solution. In this case, the optimal total potential energy $W_{t}$ of MS-MSR w.r.t. the state variables $\xi_{i}$ could be expressed as:

$$\begin{array}{c} \begin{array}{l} \underset{\xi_{i}}{\text{min}}W_{t}\left(\xi_{i}\right)=W_{m}\left(\xi_{i}\right)+W_{s}\left(\xi_{i}\right)+W_{g}\left(\xi_{i}\right)+W_{v}\left(\xi_{i}\right)\\ s.t.\left\{\begin{array}{c} {\overset{\cdot }{n}}_{i}\left(s\right)=-f_{i}\left(s\right)-I_{i}\left(s\right)\\ {\overset{\cdot }{m}}_{i}\left(s\right)=-{\overset{\cdot }{p}}_{i}\left(s\right)\times n_{i}\left(s\right)-l_{i}\left(s\right) \end{array}\right. \end{array} \end{array}$$ (15)

where $W_{m}\left(\xi_{i}\right)$, $W_{e}\left(\xi_{i}\right)$, $W_{g}\left(\xi_{i}\right)$, and $W_{v}\left(\xi_{i}\right)$ are the potential energy of the magnetic field, the elasticity of the soft beam, gravity, and blood vessel interaction of the *i*th tube (including the friction), respectively. Then, the magnetic potential energy per length $u\left(s,\;t\right)$ could be expressed as:

$$W_{m}\left(\xi_{i}\right)=-m_{E}\cdot B_{m}$$ (16)

where the dipole moment $m_{E}$ of ITM is $m_{E}=-\mu_{0}u\left(s,\;t\right)$ with magnet permeability $\mu_{0}=4\pi \times 10^{-7}\;T\cdot {\mathrm{mA}}^{-1}$. The elastic potential energy of MS-MSR is mainly from the strain energy stored in its bending and torsion configuration of the *i*th tube in a quasi-static state, which is expressed as follows:

$$\begin{array}{c} \begin{array}{c} W_{s}\left(\xi_{i}\right)=\frac{1}{2}{\left(u_{i}\left(s\right)-{\hat{u}}_{i}\left(s\right)\right)}^{T}K_{\mathrm{bt}}\left(u_{i}\left(s\right)-{\hat{u}}_{i}\left(s\right)\right)\\ \;+\frac{1}{2}{\left(v_{i}\left(s\right)-{\hat{v}}_{i}\left(s\right)\right)}^{T}K_{\mathrm{se}}\left(v_{i}\left(s\right)-{\hat{v}}_{i}\left(s\right)\right) \end{array} \end{array}$$ (17)

where $K_{\mathrm{se}}$ and $K_{\mathrm{bt}}$ are shear and bending matrices of MS-MSR, respectively. The gravitational term of the total potential energy could be derived as follows:

$$W_{g}\left(\xi_{i}\right)=\int_{0}^{\Delta l_{i}}-\rho_{i}A_{i}g^{T}p_{i}\left(s\right)\mathrm{ds}$$ (18)

where g is the gravitational acceleration that integrates the terms alongside the arc length $s_{i}$. In the scenario of intravascular intervention, MS-MSR would interact with the vasculature boundaries. Therefore, the potential energy $W_{g}\left(\xi_{i}\right)$ and $W_{v}\left(\xi_{i}\right)$ can be counteracted while conducting the intraoperative computation, i.e., the MSR is supported and interacts with the surrounding tissues. To validate the proposed method, we also compute the deflection of MS-MSR using finite element method (FEM) through the FEM software (COMSOL Inc., Stockholm, Sweden) with approximately 24,000 trigonal elements for each configuration to determine a stable MSR poses under a magnetic field $B_{m}$ with different orientations, as shown in Fig. 2D. Compared with the FEM method, the proposed optimal method could provide a mean error of 2.52±0.98° w.r.t. the ground truth.

### Mobile magnetic manipulation

**Fig. 3.**
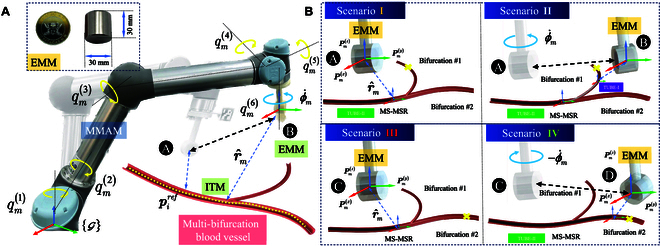
Modeling of the interaction between MMAM and MS-MSR considering the dynamic motion. (A) Pose of MMAM w.r.t. the preplanned trajectory of MS-MSR. (B) Effective phase for actuating the MS-MSR’s steering motion considering the optimized pose planning of MMAM, which is classified into 4 scenarios: Scenario I, MS-MSR is targeted for steering into a smaller bifurcation; Scenario II, MMAM rotates in a velocity of ${\overset{\cdot }{\phi }}_{m}$, while Tube-I is steered forward; Scenario III, MS-MSR is targeted for steering into a larger bifurcation; Scenario IV, MMAM rotates in a velocity of $-\;{\overset{\cdot }{\phi }}_{m}$, while Tube-I is steered forward.

The external actuation source to deflect MS-MSR is magnetic wrench $W_{m}=\left[F_{m}\left(r_{m}\right),\;T_{m}\left(r_{m}\right)\right]\in {\mathbb{R}}^{6}$, generated by the external magnetic field (see Fig. 3). We select the EMM as a spherical permanent magnet (diameter: 30 mm, NdFeB, N42 grade), which generates a heterogeneous magnetic field $B_{m}$. Considering the constraint environment of intravascular intervention, the *i*th tube’s tip trajectory $p_{i}^{\mathrm{ref}}$ of MS-MSR should be preplanned. Unlike the cable-driven soft robots, the tip motion of MSR is determined by the deflection of the magnetic wrench and the advancement of the linear advancer. Hence, the planning of the tip trajectory would partially transfer to the planning of the EMM, i.e., the 6-DOF pose $p_{m}=\left[p_{m}^{\left(x\right)},\;p_{m}^{\left(y\right)},\;p_{m}^{\left(z\right)},\;\alpha_{m},\;\beta_{m},\;\phi_{m}\right]$ of MMAM. As shown in Fig. 3A, the 3-dimensional (3D) deflection of the MS-MSR’s tip would be desired to follow the desired preplanned trajectory, especially inside the intravascular environment.

MMAM could be simplified as a rotating magnet field with a single permanent magnet. Therefore, the heterogeneous magnetic field $B_{m}$ near the distance of ${\hat{r}}_{m}$ could be expressed as follows:

$$B_{m}\left({\hat{r}}_{m}\right)=\left[\begin{array}{c} B_{m}^{x}\\ B_{m}^{y}\\ B_{m}^{z} \end{array}\right]=\frac{\mu_{0}Z}{4\pi {\left\|{\hat{r}}_{m}\right\|}^{3}}m_{E}$$ (19)

where $Z=I-5{\hat{r}}_{m}{\hat{r}}_{m}^{T}$ and I is the identity matrix. ${\hat{r}}_{m}=\left\|{\hat{p}}_{m}-{\hat{p}}_{i}\right\|$ is the norm of magnetic actuation distance. By modeling the interaction of ITM and EMM, the magnetic wrench $W_{m}=\left[F_{m}\left(r_{m}\right),\;T_{m}\left(r_{m}\right)\right]\in {\mathbb{R}}^{6}$ (including magnetic forces and torques) can be expressed as:

$$\begin{array}{c} F_{m}({\hat{r}}_{m})=(m_{E}\cdot \nabla )B_{m}\\ =\frac{3M}{{\left\|{\hat{r}}_{m}\right\|}^{4}}\left({\hat{m}}_{E}{\hat{m}}_{I}^{T}+{\hat{m}}_{I}{\hat{m}}_{E}^{T}+\left({\hat{m}}_{I}^{T}Z{\hat{m}}_{E}\right)I\right){\hat{r}}_{m} \end{array}$$ (20)

$$\begin{array}{c} T_{m}\left({\hat{r}}_{m}\right)=m_{E}\times B_{m}\\ \;=\frac{3M}{{\left\|{\hat{r}}_{m}\right\|}^{3}}{\hat{m}}_{I}\times D{\hat{m}}_{E} \end{array}$$ (21)

where $D=I-3{\hat{r}}_{m}{\hat{r}}_{m}^{T}$. In addition, ${\hat{m}}_{I}$ and ${\hat{m}}_{E}$ are magnetic moments of ITM and EMM [28], respectively. Considering that the EMM would generate attractive and repulsive forces for ITM, the planning of the EMM trajectory should follow the optimization problem to provide the minimum force principle. Here, we define the rotation angular velocity ${\overset{\cdot }{\phi }}_{m}$ of the EMM w.r.t. the *z* axis of the EE of MMAM, which is perpendicular to the permanent magnet’s axis. As shown in Fig. 3B, by utilizing MS-MSR, the steering scenarios encountering the multi-bifurcation could be classified into 4 stages as follows:

Scenario I: When the targeted bifurcation for MS-MSR has a smaller ID compared to the current one, MS-MSR is desired to steer Tube-I with opposite magnetization, MLAM should steer Tube-I forward, while the EMM should execute the corresponding motion.

Scenario II: When Tube-I of MS-MSR steers forward, MMAM should provide a sufficient magnetic field to support the adequate bending angle. The EMM rotates in an angular velocity ${\overset{\cdot }{\phi }}_{m}$ (clockwise direction) to provide the deflection magnetic force and torques and moves from position A to B.

Scenario III: When the targeted bifurcation for MS-MSR has an equivalent or larger ID compared to the current one, MS-MSR is desired to steer Tube-II without the telescoping motion, MLAM should steer Tube-II toward the targeted position, while the EMM should execute the corresponding motion.

Scenario IV: When Tube-I of MS-MSR steers forward, MMAM should provide a sufficient magnetic field to support the adequate bending angle. The EMM rotates in an angular velocity $-\;{\overset{\cdot }{\phi }}_{m}$ (counterclockwise direction) to provide the deflection magnetic force and torques and moves from position C to D.

Hereafter, the trajectory planning method of MMAM is largely based on the state of the EMM and the corresponding desired trajectory of MS-MSR, which could be transferred to the optimal problem by minimizing the interaction forces between ITM and EMM as follows:

$$\begin{array}{c} \begin{array}{l} \underset{r_{m},\phi_{m}}{\text{arg}\text{min}}\;\left|F_{i}\left(r_{m}\right)\right|=\left|\frac{1}{2\pi }\int_{0}^{2\pi }F_{m}{\left(r_{m}\right)}^{T}{\overset{\cdot }{\phi }}_{m}d\phi_{m}\right|\\ s.t.\left\|r_{m}\right\|\geq \left\|r_{s}\right\|,\phi_{m}\in \left(-\pi ,\pi \right) \end{array} \end{array}$$ (22)

where $\left\|r_{s}\right\|$ is the norm of safe interaction distance between ITM and EMM by considering the real surgical environment to protect against the collision harm of the MMAM motion.

### Analytical modeling of CMNS

In this section, we introduce the analytic modeling of CMNS to facilitate the actuation of MS-MSR during the interventional procedure. For the whole procedure, the 2 manipulators, i.e., MMAM and MUNM, are responsible for different tasks, namely, magnetic actuation and US tracking. Thus, the controller for CMNS is a task-space controller as the 2 manipulators should cooperate by considering the EEs’ motion and collision avoidance, achieving multi-objective complex tasks under different constraints [40]. Hierarchical tasks can be executed by exploiting the redundancy of a multirobot system and giving different priorities to the multi-objective tasks, which could be described as a QP problem.

**Fig. 4.**
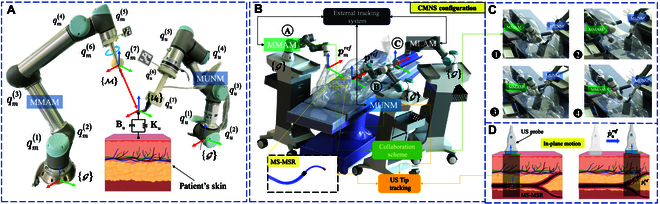
The collaborative scheme of CMNS. (A) Kinematic representation of the dual-manipulator MMAM and MUNM. (B) Whole system of CMNS with the EE of each manipulator tracked using the external tracking system via the marker to calibrate them with the global frame $\left\{G\right\}$. (C) Four kinematic collaborative scenarios of MMAM and MUNM during the interventional procedure. (D) US tracking imaging plane updating through the endovascular intervention of MS-MSR along with the movement of MUNM.

In CMNS, the multi-objective tasks are magnetic actuation and US tracking, which are executed by MMAM and MUNM, respectively. Herein, we focus on the kinematic interaction between MMAM and MUNM, as MLAM is mainly utilized to adjust the steering pose at the beginning of the intervention procedure, which could be neglected. As shown in Fig. 4, both MMAM and MUNM are 6-DOF manipulators with 6 motorized revolute joints $q_{m}^{\left(k\right)},q_{u}^{\left(k\right)}\left(k=1,\;2,\ldots ,\;6\right)$ and functional EEs as the last joint (k=7). Therefore, CMNS forms a dual-manipulator robotic configuration with different EE frames $\left\{ℳ\right\}$ and $\left\{U_{t}\right\}$ with a shared workspace $\left\{V_{m/u}\right\}$, i.e., the space above the MS-MSR’s trajectory $p_{i}\in {\mathbb{R}}^{3}$ beneath the patient’s tissue during the interventional procedure (see Fig. 4A). The transformation from the EE to the global base flame of MMAM and MUNM could be modeled as:

$$p_{m/u}\left(q_{1},\ldots ,\;q_{k}\right)=\left(\prod_{k=1}^{7}A_{k}^{k-1}\left(q_{m/u}^{k}\right)\right)$$ (23)

where $A_{k}^{k-1}\left(q_{m/u}^{k}\right)$ is a 4×4 homogeneous transformation defined by the corresponding Denavit–Hartenberg (DH) parameters of MMAM or MUNM [41]. To exploit the redundancy of CMNS while performing the multi-objective tasks, we consider the dual-manipulator as a system with 12-DOF. Then, the generalized coordinates of the multi-DOF manipulator’s joint $q_{m/u}\in {\mathbb{R}}^{12}$ define the relative pose of the EE frame. By harnessing the IK of CMNS, the desired relative task-space velocities of the 2 manipulators could be affixed to the corresponding joint space as follows:

$${\overset{\cdot }{p}}_{m/u}=J_{r}{\overset{\cdot }{q}}_{m/u}$$ (24)

where $J_{r}$ is the relative Jacobian matrix between the dual manipulators. ${\overset{\cdot }{p}}_{m/u}={\left[\begin{array}{cc} v_{r}^{1} & \omega_{r}^{1} \end{array}\right]}^{T}$ represents the relative linear and angular velocity of the EEs. $q_{m/u}={\left[\begin{array}{cc} q_{m} & q_{u} \end{array}\right]}^{T}$ is the relative joint space vector. As shown in Fig. 4B, the optical marker-based tracking method links the 2 manipulators into the global frame $\left\{G\right\}$ to calibrate the collaborative motions. For a redundant collaborative system, i.e., CMNS, various configurations of the 2 manipulators would satisfy the task requirement, which consists of minimizing the Euclidean norm of Eq. 24. The IK problem of QP of CMNS to obtain the desired ${\overset{\cdot }{q}}_{m/u}$ could be modeled as:

$$\begin{array}{c} \underset{\overset{\cdot }{q}}{\text{min}}\begin{array}{c} \left\|J_{r}{\overset{\cdot }{q}}_{m/u}-{\overset{\cdot }{p}}_{m/u}\right\| \end{array}\\ s.t.\;A{\overset{\cdot }{q}}_{m/u}=b,C{\overset{\cdot }{q}}_{m/u}\leq d \end{array}$$ (25)

where A and C are the dynamic coefficient matrices, while b and d are the constant vectors in the equality and inequality constraints. By solving the optimization QP problem of Eq. 25, we could obtain the optimal joint velocity ${\overset{\cdot }{q}}_{m/u}^{*}={\left[\begin{array}{cc} {\overset{\cdot }{q}}_{m}^{*} & {\overset{\cdot }{q}}_{u}^{*} \end{array}\right]}^{T}$. Besides, we apply an external tracking system to calibrate the multirobot system through the markers on the EEs, as shown in Fig. 4B, facilitating the uniform coordinate representation of CMNS.

*Constraints:* To prevent CMNS from exceeding any joint limits during the navigation procedure, the joint constraints in terms of position $q_{m/u}$ and velocity ${\overset{\cdot }{q}}_{m/u}$ are defined to prevent CMNS from reaching the upper and lower limits as follows:

$$q_{m/u}^{\min}\leq q_{m/u}\left(t-\Delta t\right)+{\overset{\cdot }{q}}_{m/u}\left(t\right)\Delta t\leq q_{m/u}^{\max}$$ (26)

$${\overset{\cdot }{q}}_{m/u}^{\min}\leq {\overset{\cdot }{q}}_{m/u}\leq {\overset{\cdot }{q}}_{m/u}^{\max}$$ (27)

with QP time step Δt. Apart from the joint limits, there are several scenarios in which we need to consider the velocity of MMAM and MUNM to avoid collisions (see Fig. 4C): (a) The initial position of MMAM should leave the workspace $\left\{V_{m/u}\right\}$ facilitating the initial tracking of MUNM’s motion. The initial velocity of MUNM should coincide with the MS-MSR’s steering velocity. (b) When MMAM is behind MUNM alongside the navigation direction, the velocity of MUNM should be faster than MMAM in case of potential collision. (c) When MS-MSR needs to select a bifurcation, MMAM actuates MS-MSR, and the velocity of MUNM could be slowed down. (d) When the successful bifurcation selection is achieved, MMAM should move away to leave the workspace $\left\{V_{m/u}\right\}$, and MUNM should keep tracking the MS-MSR’s tip again at the velocity of $p_{u}^{\mathrm{ref}}$.

### Model-based dynamic control

The dynamic control of CMNS could be regarded as the controller for a single manipulator, which involves a redundant system with multi-objective tasks and robot–patient interaction. It is enforced by projecting the secondary task into the null space of the primary one. The general form of the dynamic controller of CMNS is derived as follows:

$$M_{c}{\ddot{q}}_{m/u}+C_{c}\left(q_{m/u},{\overset{\cdot }{q}}_{m/u}\right){\overset{\cdot }{q}}_{m/u}+g_{c}\left(q_{m/u}\right)=\tau_{m/u}+\tau_{e}$$ (28)

where $M_{c}$, $C_{c}$, and $g_{c}$ are the inertia matrix, Coriolis and centrifugal matrix, and gravity matrix of CMNS, respectively. $\tau_{m/u}={\left[\tau_{m},\;\tau_{u}\right]}^{T}$ are the control inputs for CMNS, and $\tau_{e}$ is the external torques. To ensure the compliant behavior of the human–robot interaction, MUNM should include the interaction between the US probe and the human tissue, which is a stiffness-damping system as shown in Fig. 4A. In view of an impedance controller, we can derive the following expression for interaction force $F_{u}$:

$$M_{u}\left({\ddot{p}}_{u}^{\mathrm{ref}}-{\ddot{p}}_{u}\right)+B_{u}\left({\overset{\cdot }{p}}_{u}^{\mathrm{ref}}-{\overset{\cdot }{p}}_{u}\right)+K_{u}\left(p_{u}^{\mathrm{ref}}-p_{u}\right)=F_{u}$$ (29)

where $M_{u}$ is the inertial matrix, $B_{u}$ is the damping matrix, and $K_{u}$ is the stiffness matrix between the US probe and human tissue. By utilizing Eq. 24, we can control the dual-manipulator system as a single manipulator with redundancy from the control perspective. Then, the overall controller contains the null-space controller, which can be expressed as follows:

$$\tau_{u}^{c}=J_{u}^{T}F_{u}+\tau_{n}^{\left(1\right)}+\tau_{n}^{\left(2\right)}$$ (30)

where $J_{u}$ is the Jacobian matrix of MUNM. The first null-space controller involves the synchronization of MUNM and MS-MSR, as shown in Fig. 4D. The imaging features $s_{\mathrm{ui}}=\left[u_{i},\;v_{i}\right]$ of MS-MSR are constrained to keep the MS-MSR’s tip inside the field of view (FOV) of the US probe, which could be defined as the *Visibility constraint* as follows:

$$\left[\begin{array}{c} u_{\min}\\ v_{\min} \end{array}\right]\leq s_{\mathrm{ui}}\leq \left[\begin{array}{c} u_{\max}\\ v_{\max} \end{array}\right]$$ (31)

where $u_{\min}$ and $v_{\min}$ ($u_{\max}$ and $v_{\max}$) represent the minimum (maximum) horizontal and vertical boundaries of US image feature in pixels. Then, the corresponding cost function of the US probe velocity is derived as:

$$\begin{array}{l} \underset{s_{\mathrm{ui}}}{\text{min}}\left\|{\overset{\cdot }{p}}_{u}^{\mathrm{ref}}-k_{p}^{u}L_{u}\left(s_{\mathrm{ui}}^{\mathrm{ref}}-s_{\mathrm{ui}}\right)\right\|\\ s.t.\text{Visibility}\;\text{constra}\mathrm{int} \end{array}$$ (32)

where $k_{p}^{u}$ is the proportional gain for the US probe controller and $L_{u}$ is the US interaction matrix. Hence, the first null-space controller is expressed as follows:

$$\tau_{n}^{\left(1\right)}=C\left(q_{u},\;{\overset{\cdot }{q}}_{u}\right)J_{u}^{T}L_{u}\left[N_{t}^{b}{\left({\overset{\cdot }{p}}_{i}\right)}^{-1}J_{i}\right]+H_{\mathrm{dyn}}\left(q_{u},\;{\ddot{q}}_{u}\right)$$ (33)

where $N_{t}^{b}$ is the transform matrix from the MS-MSR’s tip to the base, $J_{i}$ is the Jacobian matrix defined in Eq. 1, and $H_{\mathrm{dyn}}\left(q_{u},\;{\ddot{q}}_{u}\right)$ is the dynamic term of MUNM manipulator. For the second null-space controller, the QP-based controller could be expressed as a cost function by adding the control gain to Eq. 25 as follows:

$$\begin{array}{c} \begin{array}{l} \underset{{\overset{\cdot }{q}}_{m/u}}{\text{min}}\begin{array}{c} \left\|J_{r}{\overset{\cdot }{q}}_{m/u}-\left({\overset{\cdot }{p}}_{m/u}^{\mathrm{ref}}+K_{p}\left(p_{m/u}^{\mathrm{ref}}-p_{m/u}\right)\right)\right\| \end{array}\\ s.t.\;A{\overset{\cdot }{q}}_{m/u}=b,C{\overset{\cdot }{q}}_{m/u}\leq d \end{array} \end{array}$$ (34)

where $K_{p}$ is the proportional gain of CMNS and $\tau_{n}^{\left(2\right)}$ could be calculated using the general dynamics of Eq. 28. The overall controller is depicted in Fig. 5.

**Fig. 5.**
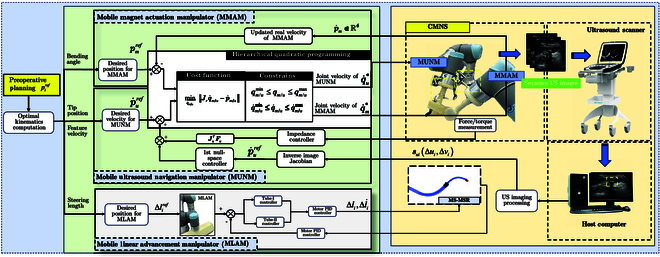
The control block diagram of CMNS and MS-MSR using the optimization method. This controller addresses the collaboration motions of the multirobot system with constraints.

## Results

### Experimental setup

We validated the proposed analytical modeling and the performance of the proposed MS-MSR and CMNS in terms of steering accuracy, kinematic maneuverability, dynamic responses, and the capability of collaboration. The experimental apparatus is deployed on an experimental optical table. The host computer is an off-the-shelf Intel NUC equipped with an Intel Core i7-11700 @2.60 GHz with 32 GB of RAM. The MS-MSR’s codebase is MATLAB for model solving, while the CMNS’s code is mainly written by Python 3.7/C++ in Robot Operation System (ROS Noetic). The experimental flow is presented as follows. First, the kinematic modeling and dynamic characteristics of MS-MSR were verified along with the free-end deflection test. Then, a follow-the-lead dexterity test of MS-MSR inside a 3D sequential-ring scene is conducted. Finally, we conduct in vitro experiments to steer MS-MSR through a biological gelatinous phantom with the guidance of CMNS.

### Free-fixed bending kinematics and dynamic responses

**Fig. 6.**
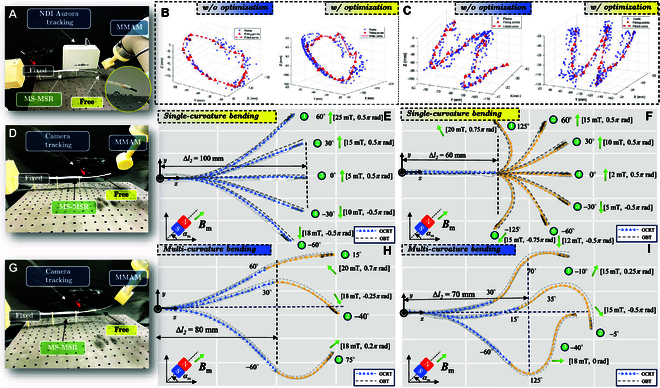
Testing of kinematic and dynamic responses of MS-MSR with free-bending configuration. (A) Experimental setup with NDI Aurora tracking system to perform the dynamic tip following test with the Tube-II base fixed at $p_{2}\left(s_{2}=0\right)={\left[0,\;0,\;0\right]}^{T}$. (B) Dynamic performance of MS-MSR following preset tip-space trajectories of a circle w/ (right) and w/o (left) optimization scenario. (C) Dynamic performance of MS-MSR following preset tip-space trajectories of letter “W” under w/ (right) and w/o (left) optimization scenario. (D to F) Single-curvature bending (SCB): Quasi-static SCB scenario with different bending angle of MS-MSR by the magnetic field $B_{m}$ provided by MMAM with different tube length, i.e., $\left[\Delta l_{1}=0,\;\Delta l_{2}=100\mathrm{mm}\right]$ and $\left[\Delta l_{1}=60\mathrm{mm},\;\Delta l_{2}=60\mathrm{mm}\right]$. (G to I) Multi-curvature bending (MCB): Quasi-static multiple curvature bending scenario of MS-MSR with different tube length, i.e., $\left[\Delta l_{1}=80\mathrm{mm},\;\Delta l_{2}=60\mathrm{mm}\right]$ and $\left[\Delta l_{1}=80\mathrm{mm},\;\Delta l_{2}=70\mathrm{mm}\right]$.

As shown in Fig. 6, to quantitatively evaluate the kinematic and dynamic characteristics of MS-MSR, the force–deflection relation with proximal end fixed was tested. The predicted shape of MS-MSR is based on the optimized Cosserat rod theory proposed in the previous section. The specifications of MS-MSR are given in Table 1 with Young’s modulus of 2.4 MPa. Herein, we set the proximal end of Tube-II fixed in both position and orientation as the deflection origin, i.e., $p_{2}\left(s_{2}=0\right)={\left[0,\;0,\;0\right]}^{T}$. Two sets of experiments were conducted to reveal the characteristics of MS-MSR in 2 states, i.e., dynamic and quasi-static states.

First, the dynamic response of MS-MSR is tested through the predefined trajectory tracking test. The distal tip trajectory of the *i*th tube $p_{i}\in {\mathbb{R}}^{3}$ is recorded by the NDI Aurora tracking system (Northern Digital Inc., Ontario, Canada) (see Fig. 6A). Herein, Tube-II (with a free-end length of 65 mm) would follow a spatial 3D circle tip trajectory with a diameter of 100 mm and a letter trajectory “W” of the size of 100mm×100mm. The test results are shown in Fig. 6B. The results demonstrate that MS-MSR could stably track the reference trajectory with mean tracking errors $\varepsilon_{i}$ of 1.32 and 1.86 mm for each tip trajectory, with a standard deviation (SD) of 0.45 mm. As compared with other beam theory calculation, the proposed method shows a higher accuracy. The dynamic response of MS-MSR is fast, with an average completion time of 3.22 and 4.56 s for each tip trajectory, which validates the theoretical model (the PDEs) derived in the previous section.

Second, the quasi-static bending performance of MS-MSR under the external magnetic field $B_{m}\in {\mathbb{R}}^{3}$ is tested to reveal its shape prediction accuracy with an average 30 calculated distributed points, as shown in Fig. 6D to I. A stereo camera (model: ZED 2i, from Stereolabs, CA, USA) is utilized to obtain the shape ground truth of MS-MSR. In this test, MS-MSR is actively bent within the plane perpendicular to the optical platform and maintains quasi-static states during data recording. The MS-MSR’s gravity is considered in the experimental validations. We classify the tests into 2 categories, i.e., single-curvature bending (SCB) and multi-curvature bending (MCB). Here, we set the external magnetic field, i.e., $B_{m}\in {\mathbb{R}}^{3}$, with different magnitudes and orientations. In the first scenario of SCB, the proximal end of Tube-II is fixed. We set the transmission length of Tube-I and Tube-II as $\left[\Delta l_{1}=0,\;\Delta l_{2}=100\;\mathrm{mm}\right]$ (see Fig. 6E), i.e., without the telescopic state. The testing results indicate that the bending angle range, in this case, is from −60° to 60° (could be large with a stronger magnetic field) with an average shape prediction error of 3.47±1.02mm and a tip tracking error of 1.68±0.65mm as shown in Table 2. In the second scenario of SCB, MS-MSR is in the telescoping state with $\left[\Delta l_{1}=60\;\mathrm{mm},\;\Delta l_{2}=60\;\mathrm{mm}\right]$ (see Fig. 6F). Here, we fix Tube-II at both the proximal and distal ends to show the separate movement of Tube-I. The testing bending angle is from −125° to 125° with an average shape prediction error of 2.27±1.02mm and a tip tracking error of 1.50±0.42 mm. In the first scenario of MCB, MS-MSR is in the telescoping state with $\left[\Delta l_{1}=80\;\mathrm{mm},\;\Delta l_{2}=60\;\mathrm{mm}\right]$ (see Fig. 6H). Tube-I and Tube-II were in different bending states, e.g., $\left[-\;60{}^{\circ},\;75{}^{\circ}\right]$. In the second scenario of MCB, MS-MSR was in the telescoping state with $\left[\Delta l_{1}=80\;\mathrm{mm},\;\Delta l_{2}=70\;\mathrm{mm}\right]$ (see Fig. 6I), which would facilitate the MCB scenario of intravascular intervention, e.g., $\left[30{}^{\circ},\;70{}^{\circ},\;-\;10{}^{\circ}\right]$. It is observed that the experimental results are consistent with the results of theoretical analysis in the previous section, with an overall mean shape error of 3.62° and a tip error of 1.08 mm. The results reveal that MS-MSR can achieve a large range of bending curvature and high accuracy of shape error and tip error using the proposed optimized Cosserat rod theory.

**Table 2.** Comparison results of optimized Cosserat rod theory and conventional beam theory

Case	#No.	Bending	Shape error °			Tip error [mm]		
			Mean	SD	OBT ^a^	Mean	SD	OBT
SCB	1	[0, 60°]	3.67	±1.17	5.45	1.18	±0.65	2.45
	2	[0, 30°]	3.86	±1.22	5.24	1.22	±0.34	2.24
	3	[0°, 0°]	3.63	±1.35	4.87	1.28	±0.43	2.33
	4	[0°, −30°]	3.79	±1.15	4.57	1.23	±0.32	1.97
	5	[0°, −60°]	3,84	±1.12	4.34	1.25	±0.53	2.29
SCB	1	[125°, 0°]	3.36	±1.43	4.28	1.38	±0.49	2.36
	2	[75°, 0°]	3.27	±1.11	4.27	1.34	±0.36	2.57
	3	[30°, 0°]	3.12	±1.32	5.03	0.94	±0.38	2.82
	4	[0°, 0°]	3.15	±1.08	4.22	0.97	±0.45	2.65
	5	[−30°, 0°]	3.18	±1.24	4.56	1.12	±0.53	1.93
	6	[−75°, 0°]	3.35	±0.92	5.37	1.05	±0.38	2.58
	7	[−125°, 0°]	3.27	±1.45	5.25	1.10	±0.42	2.87
MCB	1	[60°, 15°]	3.46	±1.02	4.24	1.34	±0.48	2.85
	2	[30°, −40°]	3.56	±1.32	6.07	1.43	±0.39	2.06
	3	[−60°, 75°]	3.67	±1.25	4.78	1.22	±0.45	2.56
MCB	1	[30°, 75°, −10°]	3.53	±1.18	5.21	1.42	±0.47	2.22
	2	[15°, 35, −5°]	3.55	±1.35	4.89	0.89	±0.53	2.64
	3	[−60°, 125°, −40°]	3.67	±1.33	4.82	1.13	±0.55	2.77

^a^ Other beam theory

### Tortuous path of sequential ring-steering test

**Fig. 7.**
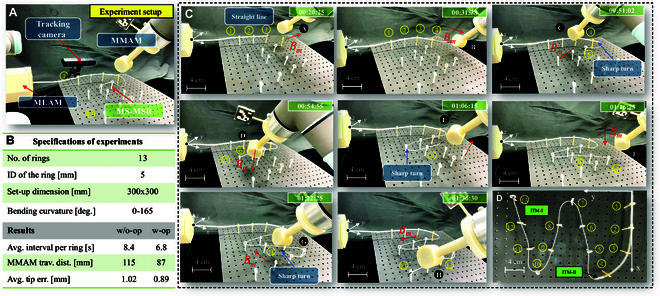
Demonstration of autonomous intervention and navigation capabilities of MS-MSR through a 3D tortuous path (13 rings with the shape of the letter “M”). (A) Experimental setup with MMAM and MS-MSR with a large orientation difference between adjacent rings. (B) Specifications of tests and results. (C) Snapshots of the ring-steering tests: The motion of MS-MSR’s tip $p_{i}\in {\mathbb{R}}^{3}$ is the superposition of bending and steering, actuated by the magnetic field $B_{m}$ provided by MMAM and insertion motion by MLAM. The magnetic robotic soft robot is required to pass through the center of the ring set $p_{i}^{\mathrm{ref}}$ with different central positions, heights, and orientations. (D) Final steering results of MS-MSR.

As MS-MSR could achieve MCB, the kinematic response and dynamic dexterity of MS-MSR are further validated by the tests of maneuvering through a sequence of 3D rings mimicking the constraint and sophisticated environment, e.g., the intravascular environment, navigated by the rotating magnetic field $B_{m}$ of MMAM. As shown in Fig. 7A, a sequence of rings (ring number = 13, ID = 5 mm, height = 50 mm) is vertically placed on a 300×300mm polyphonous test board with variable-centric positions and orientations, forming the trajectory of the letter “M”. The tortuous path simulates the navigation waypoints during the intravascular procedure, e.g., from the femoral entry to the aorta arch. The basic principle for this test is to align the central axis (denoted *z* axis upward) of ITM and the soft rod along the desired direction to create bending of the MS-MSR’s distal end toward the reference direction as preplanned presented in the previous section by controlling the rotating magnetic field **B**_m_ and the linear advancement module. MS-MSR would telescope Tube-I forward when encountering a larger curvature bending scenario and sharp turn (>90°), as shown in CASE-C-E-G in Fig. 7C. We adopt a stereo camera ZED 2i (Stereolabs, CA, USA) to record the motion of MS-MSR and MMAM.

From Fig. 7B, we can see the test results with the optimal method and without the optimal method comparisons for both MS-MSR and MMAM. The experimental snapshots are shown in Fig. 7C. The average interval between 2 adjacent rings is 6.8 s compared to the method without optimization of 8.6 s, which indicates a higher steering accuracy with fewer contact trials. MMAM conducts less traveling distance between each ring completion using the optimal method proposed in the previous section. The test results show that MS-MSR could precisely navigate itself through the 3D multi-ring configuration and reach the target position with no missed steering (see Fig. 7D). The average steering completion time is 96.50±2.42 s within 10 experimental trials, compared to the method without optimization of 124.15±4.57 s. The test results reveal that the proposed optimal modeling and actuation method for MS-MSR has a higher dexterity and accuracy than conventional methods.

### Steering test within a multi-bifurcation phantom

**Fig. 8.**
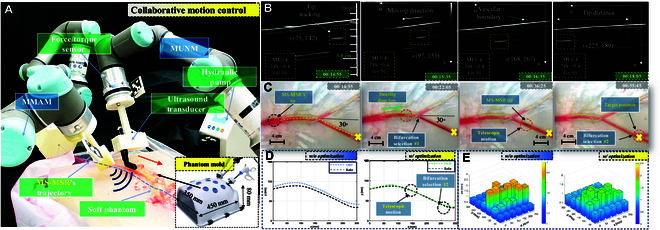
In vitro intravascular testing under magnetic actuation and US tracking of MS-MSR with soft anthropomorphic phantom. (A) Experimental setup for multirobot actuation and tracking system. (B) Real-time tip tracking (yellow rectangle) of US probe (16HL7) with different tracking frames with the selected US parameters: thermal index score (TIS) < 0.4, mechanical index (MI) of 0.4, gain settings (Gn) of 50, frequency of 7 to 16 MHz, and depth of 5.0 cm. (C) The trajectory following scenario of 2 bifurcation-selection of MS-MSR. (D) Intraoperative shape estimation using the proposed optimal Cosserat rod modeling compared with other beam theories. (E) The error color map comparisons of the proposed method with the optimization.

To validate the analytical modeling effectiveness of the proposed MS-MSR along with CMNS within the intravascular environment, an in vitro autonomous steering test is conducted with intraoperative magnetic actuation and US tracking within a shared workspace $\left\{V_{m/u}\right\}$, while MS-MSR is steered within a multi-bifurcation vascular phantom beneath the soft tissue, as shown in Fig. 8A. The experimental phantom is fabricated as a soft half-transparent gelatinous phantom molded from a laparoscopic surgical training model (size: 450×350×80mm) with a multi-bifurcation vascular phantom model embedded (2.2≤ID≤8.6mm). MUNM is equipped with an L-shape US probe 16HL7 (7 to 16 MHz) through a 3D-printed holder connected to a US scanner (uSmart 3300, Terason, MA, USA) using the B-mode imaging streaming (size of 256×300 pixels). A 6-DOF force/torque sensor (HEX, Onrobot, Denmark) is utilized for the interaction force measurement. The MS-MSR’s tip is intraoperatively tracked using the optimized visual-based tracking algorithm proposed in the previous section during the US probe continuous scanning, as is shown in Fig. 8B. The control frequency of CMNS is set as 125 Hz, which coincides with the working frequency of the UR3e/5e robotic manipulator. The cardiovascular blood fluid system is simulated by connecting the phantom with a hydraulic pump with a fluid velocity of 35 ml/min pseudo-blood flowing. We also exploit a stereo camera ZED 2i (Stereolabs, CA, USA) to connect the frames of each manipulator (i.e., MMAM and MUNM) by ArUco markers, as presented in Fig. 4B. The controller parameters for CMNS are listed in Table 3. 

The depth, frequency, and dynamic range of the US images are set as 5.0 cm, 29 Hz, and 66 dB, respectively. Herein, we track the MS-MSR’s tip using the in-plane optimal US imaging tracking method proposed in the previous section. As shown in Fig. 8B, the stable and clear US imaging of the MS-MSR’s tip and the vasculature boundaries are achieved with a tracking error of 1.52 mm. During the sequential in-plane motion of MS-MSR, the US probe could adjust its position to maintain the MS-MSR’s tip within the image frame. In this test, MS-MSR is required to follow an intravascular trajectory that has 2 bifurcation-selection scenarios (see Fig. 8C). MMAM is transformed to a start pose to incorporate the MS-MSR’s tip within the effective range of the magnetic field $B_{m}$. The intraoperative shape of MS-MSR is estimated using the proposed Cosserat rod model with a computation time of 0.23 s and a mean estimation error of 3.35° (see Fig. 8D and E), compared with the method without optimization. The experimental results show that MS-MSR could be navigated to make correct bifurcation selection utilizing the optimal strategy proposed in the previous section with an average tracking error of 1.35 mm within the least navigation time compared with the conventional ones, which is tabulated in Table 4.

**Fig. 9.**
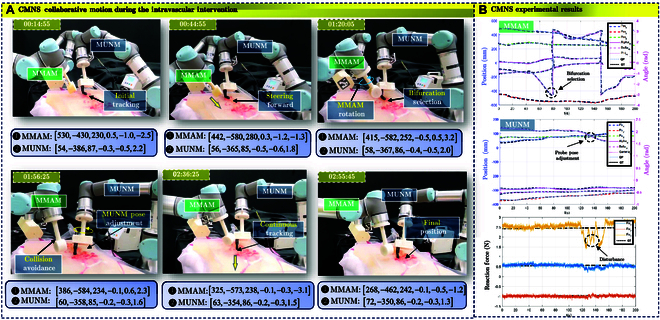
The performance of the QP-based task-space multi-objective controller for CMNS to navigate MS-MSR. (A) Motion snapshots of CMNS with MMAM (left) and MUNM (right). (B) QP-based control method under the reference trajectory of EE task-space position tracking of MMAM (top) and MUNM (middle). The reference force tracking of MUNM (bottom). Unstable tracking would occur during the scanning.

The movement snapshots of CMNS are shown in Fig. 9A, which is capable of optimizing both MMAM and MUNM motion. The working procedure is as follows. First, MUNM is actuated to acquire stable US images with a clear tip of MS-MSR, as shown in Fig. 8B. After that, MUNM and MMAM should move forward alongside the preplanned trajectories and constrained velocities shown in Fig. 5. The QP-based method regulates the motion of MMAM and MUNM by minimizing Eq. 25 using the QP solver toolbox to achieve collision avoidance (see Table 4). The tracking error in the task space for MMAM and MUNM is $\left[1.79\;\mathrm{mm},1.26\;\mathrm{rad}\right]$ and $\left[1.62\;\mathrm{mm},1.37\;\mathrm{rad}\right]$, respectively (see Fig. 8B), which is superior to the method without QP. From Table 4, we can observe more detailed results of this experiment. There is the case that the target loss (around 25.4%) of the soft robot’s tip during the scanning is much less than the other 2 methods. The proposed QP-based controller could realize the cooperative motion of the 2 manipulators without collision. The impedance control method proposed in the previous section is validated with the US probe keeping contact with the mock phantom at the probe velocity of 11.32 mm/s. The corresponding force tracking results are shown in Fig. 9B. We can see that the contact forces are stabilized around 2.46 N with a standard derivation of 1.43 N compared with the ground truth in the *z*-axis direction, which indicates the successful implementation of the proposed controller.

**Table 3.** Controller parameters list of the proposed method

Parameters	Value	Unit
Stiffness matrix $K_{u}$	*diag* [2000, 2000, 2000]	[N/m]
Damping matrix $B_{u}$	*diag* [30]	[N/(m/s)]
Time interval Δt	0.001	[s]
Gain of US probe $k_{p}^{u}$	0.7	N/A
Joint position limit $q_{m/u}^{\min},q_{m/u}^{\max}$	$\left(-\pi ,\;\pi \right)$	[rad]
Joint velocity limit ${\overset{\cdot }{q}}_{m/u}^{\min},{\overset{\cdot }{q}}_{m/u}^{\max}$	$\left(0,\;0.17\right)$	[rad/s]

**Table 4.** The test performance for MS-MSR and CMNS

MS-MSR	Value	Unit	MMAM | MUNM	Value	Unit
US error	1.52	[mm]	Initial tracking	13.0 ± 0.7	[s]
Steering velocity	11.3	[mm/s]	Multirobot collision	0	[times]
Collision with boundary	4.3	[times]	Average EE’s error	1.56 ± 0.15	[mm]
Tip estimation error	1.35	[mm]	Average joint velocity	0.12 ± 0.05	[rad/s]
Model computation time	0.23	[s]	Average probe velocity	11.32 ± 0.54	[mm/s]
Shape estimation error	3.35	[deg]	Navigation period	202.5 ± 4.6	[s]
Loss target rate	25.4	[%]	Average force	2.46 ± 1.43	[N]

## Discussion

The results of the above tests show that the proposed MS-MSR and CMNS could be beneficial during the autonomous intravascular intervention procedure. Compared with state-of-the-art MSRs and their corresponding MNSs in several typical aspects, the performance of the proposed method is demonstrated in Table 5. For the kinematics analysis, we adopted the Cosserat rod theory with dynamic analysis. Compared with other beam theories in the literature [6,7], our method could predict the shape and tip trajectory of MS-MSR with higher accuracy. Especially, the multi-section characteristic has proved to be effective in the complex multi-bifurcation endovascular environment, taking advantage of the homocentric structure. The proposed controller for CMNS brings several advantages to collaborative tasks. Unlike the previous works [27,42] that actuate the magnet soft robot by a stationary magnetic field, our work employs a multi-arm mobile robotic actuation and tracking system with a multiple-manipulator configuration, which provides a larger workspace for long-term navigation. Regarding clinical relevance, we test our method in a multi-bifurcation phantom with US feedback, which shows more potential than the camera-based methods [22]. Considering the friction and the interaction model with the vasculature tissue, a new material design of coating could be considered in the future design [43].

**Table 5.** Performance comparison of the state-of-the-art magnetic soft robots in minimally invasive surgery

Reference	Kinematic modeling	MSR structure diameter (mm)	Modeling accuracy (mm)	MNS type	MNS control	Optimization	Tracking method
[6]	Euler–Bernoulli theory	Single-section 1.4	1.79	mMNS	Current control	No	Camera US
[7]	Pseudo-rigid body	Single-section 2	2.2	sMNS	N/A	Yes	Camera
[16]	Kirchhoff rod theory	Multi-section 2	1.61 ± 0.62	mMNS	Position control	No	Camera
[17]	Cosserat rod theory	Multi-section 0.25/0.65/1.07	0.8 ± 0.22	sMNS	N/A	No	Camera
[22]	Euler–Bernoulli theory	Single-section 1.5	1.26 ± 0.74	mMNS	Position control	No	Camera
[29]	Euler–Bernoulli theory	Single-section 1.0	1.68 ± 0.3	mMNS	N/A	No	X-ray
This work	Cosserat rod (dynamic)	Multi-section 1.8/3.5	1.08 ± 0.45	mMNS	QP-based control	Yes	Camera US

Our key contribution is the successful implementation of MS-MSR with a multirobot actuation and tracking system with theoretical modeling and experimental validations. To our knowledge, no comprehensive MSR system with equivalent functionalities has been proposed in the literature. The successful validation of the proposed CMNS would enhance the assurance of the surgeon when considering replacing the traditional manual intervention method with a robotic autonomous one. In future work, we will further enhance our method by applying a high-level US imaging algorithm, e.g., deep learning-based vessel segmentation. The constant-force impedance controller for the robotic US proposed in this work is still not satisfactory, and there are still disturbances during the procedure. The constant-force mechanism [44] would be a good solution to this problem. Potential animal trials will be conducted to validate the magnetic robotic soft robot performance inside the real anatomy.

## Conclusion

In this paper, we have introduced the analytical modeling and optimization of a novel MS-MSR, which is driven by a CMNS with simultaneous magnetic actuation and US tracking. Optimized modeling of MS-MSR based on the Cosserat rod theory is proposed by minimizing the total potential energy, and the external heterogeneous magnetic field is modeled, which generates desired magnetic forces/torques while achieving the optimal planning of MMAM. We also provide a QP-based optimal controller to regularize the collaborative motion of MMAM and MUNM with multi-objective tasks to navigate MS-MSR within the vasculature. Scientific tests have been performed to validate the design and methods, which demonstrate the effectiveness of MS-MSR and CMNS performance in terms of shape prediction, dexterity, and endovascular intervention by conducting in vitro US-guided experiments. Overall, our key contribution is the effective and precise analytical modeling for both MS-MSR and CMNS. The successful validations have solidified the potential of medical applications for future intravascular procedures.

## Data Availability

All data needed to evaluate the conclusions are presented in the manuscript and/or the Supplementary Materials. Additional data that support the findings of this study are available from the corresponding author upon reasonable request.
